# Bier block as a successful management of a patient with intractable complex regional pain syndrome (CRPS) type 1: A case report

**DOI:** 10.1002/ccr3.4554

**Published:** 2021-07-21

**Authors:** Seyed Ali Emami, Hossein Majedi, Ebrahim Espahbodi, Mehdi Sanatkar

**Affiliations:** ^1^ Anesthesiology and Pain Department Imam Khomeini Medical Center Tehran University of Medical Sciences Tehran Iran; ^2^ Neuroscience Research Centre Neuroscience Institute Tehran University of Medical Sciences Tehran Iran

**Keywords:** Bier block, case report, chronic pain, complex regional pain syndrome, intravenous regional anesthesia

## Abstract

Bier block was successful in the pain management of complex regional pain syndrome (CRPS) type 1.

## Graphical abstract

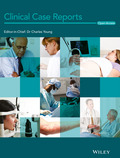

## 1 BACKGROUND

Complex regional pain syndrome is an intractable chronic pain which affects a limb or region of the body. Bier block successfully managed the pain of CRPS type 1 and had a positive impact on daily functioning, sleep, and performing routine activities.

Complex regional pain syndrome (CRPS) is a challenging chronic pain disorder identified after an aberrant response to trauma or surgery, but it can also occur spontaneously.^1^ This syndrome is characterized by exaggerated burning pain, allodynia, edema, sudomotor dysfunction, joint stiffness, and trophic changes in hair and nails.^2^ The estimated incidence of this syndrome is 5.46 to 26.2 per 100,000 people annually, and it occurs three times more often in females.^3^ There are two types of CRPS. CRPS type 1, also known as reflex sympathetic dystrophy, is not associated with nerve injury; it is identified following a major trauma, such as surgery or bone fracture, or a minor trauma, such as sprain or bruise in a limb or remote body area.^2^ CRPS type 2, also known as causalgia, involves objective injury to a specific peripheral nerve or one of its branches and is usually caused by trauma.^4^ In this syndrome, the areas of pain may not correspond to the anatomic distribution of the injured peripheral nerve. There is no single test to confirm CRPS. It is diagnosed based on history, physical examination, and differentiation from other causes. Currently, the pathophysiology of CRPS is not fully understood. The literature indicates that different pathophysiologic mechanisms are involved in CRPS, such as central and peripheral sensitization, altered sympathetic function, decreased density of C and Aδ fibers, increased inflammatory mediators, altered vasomotor function, genetic susceptibility, and psychological factors.^5^ Because multiple mechanisms are involved in CRPS, the therapeutic strategies for treating this syndrome are varied. Multidisciplinary approaches such as pain management and psychological support can be effective in improving management of these cases. It has been shown that one third of CRPS cases have achieved full recovery with current therapeutic strategies.^6^ Currently, the management of CRPS includes medications, physical, occupational, psychological therapies, and interventional modalities. However, these therapies might not be completely effective in controlling CRPS or achieving full pain relief and restoring daily functioning.^7^ Medications include nonsteroidal anti‐inflammatory drugs (NSAIDs), antidepressants, calcium channel blockers, anticonvulsants, N‐methyl‐D‐aspartate (NMDA) receptor antagonists, corticosteroids, opioids, and intravenous immunoglobulins (IVIG). Interventional therapies for the management of CRPS include stellate ganglion, brachial plexus block, lumbar sympathetic block, spinal cord stimulation, and peripheral nerve stimulation.^8^ Sympathetic block is a well‐established strategy for controlling the syndrome in these patients, but there is no specific protocol for selecting subjects who respond appropriately to this procedure.^9^ It has been shown that nerve blocks may be effective for the diagnosis and treatment of CRPS cases with severe pain, allodynia, and vasomotor changes that do not respond to pharmacotherapies.^10^ Patients who do not respond to nerve blocks may show improvement with spinal cord stimulation or peripheral nerve stimulation.^2^ Destructive sympathectomy such as radiofrequency, chemical, and surgical techniques is a controversial treatment for CRPS, because no proper response to pain control or the development of a new pain syndrome may arise after this procedure.^11^ Therefore, some studies have recommended that destructive sympathectomy be performed if nerve blocks and spinal cord stimulation fail.^10^ Intravenous regional anesthesia (Bier block) is a reliable and cost‐effective procedure for operative procedures on the extremities. It is performed on an ambulatory basis and has a success rate of 94%–98%.^12^ This paper details the treatment of a CRPS type 1 patient who had tried various therapies and interventions which were ineffective and were successfully managed with Bier block.

## 2 CASE PRESENTATION

A 36‐year‐old female patient without a past medical history was referred to a multidisciplinary pain clinic at our center for severe intractable pain in her right arm, wrist, and hand. Progressive pain developed in our case one month after orthopedic surgery on her forearm. She suffered a consistent severe stabbing and shooting pain, hyperalgesia, and allodynia with cramping and numbness in her right upper limb which interfered with her sleep. On physical examination, severe intractable pain, allodynia, hyperalgesia, skin temperature and color asymmetry, edema, and trophic changes in the nails of the right upper limb were identified. Full flexion, extension, and abduction at the right shoulder, elbow, and wrist were restricted. Neurological examination was normal. Magnetic resonance imaging, X‐ray, laboratory blood tests, and work‐up identified no pathology, thus supporting the diagnosis of CRPS. Thrombosis was ruled out through a color Doppler ultrasound. A nerve conduction study showed no significant difference between the right and left upper limbs. The patient's pain score was 10 out of 10 on the numerical rating scale (NRS), where 0 is the total absence of pain and 10 is the worst pain imaginable. Her quality of life had clearly deteriorated. CRPS type 1 was diagnosed according to the International Association for the Study of Pain (IASP) criteria.^6^ The patient took tramadol HCL 100 mg, celecoxib 200 mg, pregabalin 75 mg, and amitriptyline 25 mg, but they had no effect. She received a stellate ganglion block (SGB) with 2 ml bupivacaine HCL 0.5% and 2 ml of lidocaine 2% and reported a significant decrease in pain (NRS improved from 10 to 3) for 6 days after block. The patient tolerated this procedure well, but her pain returned after one week. As before, the pain was severe and constant and interfered with her sleep. The patient then underwent pulsed radiofrequency (PRF) of stellate ganglion one month later with RF current for 20 milliseconds at 2 Hertz and maximum electrode temperature below 42°C for a total duration of 120 seconds, but her pain again returned after one week and was severe, constant, and interfered with sleep. The patient underwent a brachial plexus block through the axillary block approach, but it was ineffective. Six months later, she received authorization for a Bier block, which she underwent with 20 ml lidocaine 0.5% and 8 mg dexamethasone. This procedure was well tolerated, and for six months after the procedure, the patient's pain decreased more than 80% with her pain score reduced to 3 on the NRS and a near‐normal performance status for daily activities.

## 3 DISCUSSION AND CONCLUSIONS

The case reported herein had severe pain, allodynia, temperature and color asymmetry, edema, and trophic changes in the fingernails of her right upper limb and met the diagnostic criteria for CRPS type 1 based on the IASP criteria.^6^ Various treatments including medications, physical therapy, and SGB did not help. Because previous studies have established that nerve blocks may improve the signs and symptoms of CRPS, a Bier block was thought to be a good treatment alternative and had a positive impact on our patient's intractable pain, sleep, and daily activities. According to previous studies, the management of patients with CRPS is complex. The literature reports that a multimodal approach is the mainstay of management in these patients. NSAIDs, antidepressants, anticonvulsants, corticosteroids, IVIG, and interventional approaches such as epidural, peripheral nerve, and various sympathetic blocks accompanied by physical therapy have some positive effects on the signs and symptoms of CRPS; however, only a few studies have been successful in treating patients with CRPS.^10^ Frequently, therapeutic regimes for CRPS are derived from the treatment of patients with neuropathic pain. Based on the latest IASP definition of neuropathic pain, however, CRPS type 1 is not included in this category.^13^ NSAIDs comprise the primary therapy started for CRPS type 1 patients before they are referred to a specialized pain clinic. The efficacy of these agents is not exactly clear in CRPS.^14^ Moreover, the evidence that other medications such as opioids, lidocaine, and botulinum toxin injections are effective in the management of severe pain in CRPS subjects is insufficient.^15^ Some studies have reported tricyclic antidepressant agents, gabapentinoid drugs, and norepinephrine serotonin reuptake inhibitor (SNRI) agents as being effective in controlling the signs and symptoms of CRPS patients.^15^ Nevertheless, many CRPS patients, including our case, are resistant to these agents. Our patient was resistant to all such medications, including celecoxib 400 mg, tramadol 200 mg, nortriptyline 25 mg, and pregabalin 150 mg daily. Based on the author's clinical experiences, SGB with bupivacaine provides an average of 5–7 days of pain relief.^16^ Ferrillo showed that SGB with liposome bupivacaine provided approximately 18–21 days of pain relief. He concluded that the administration of SGB should always be considered early in the management of CRPS, because it may help reverse progression if used proactively.^17^ SGB was performed for our patient and determined that the mean duration of pain relief with bupivacaine 0.5% HCL/lidocaine 2% was 6 days. The duration of pain relief after PRF of stellate ganglion in our case was one week. Previous studies have reported that a brachial plexus block as a single block or continuous infusion through a catheter inserted with local anesthesia and an additive such as steroids or morphine can treat the pain of CRPS patients.^18^ Wong and Wilson reported pain relief with a brachial plexus catheter using the axillary approach in a patient with severe CRPS.^19^ An interscalene block was evaluated and reported to be as effective as SGB for the management of severe pain in a patient with CRPS.^18^ Kingery used an interscalene block with an infusion pump of bupivacaine for a period of 3 to 6 months and showed good results in patients with CRPS.^20^ Fallatah reported the successful management of CRPS type 1 using a single interscalene brachial plexus block and showed dramatic improvement after one injection.^21^ The case reported herein underwent a brachial plexus block through an axillary block approach, and this procedure was ineffective. Because of the good results of various nerve blocks in the treatment of CRPS patients, we decided to use a Bier block in our patient who was resistant to medications and the SGB block. The Bier block is a reliable, easy to perform, and cost‐effective method for procedures on the extremities with success rates of 94% to 98%.^12^ Various adjutants such as opioids and steroids have been added to local anesthesia to improve block reality.^12^ There are different suggested sites for the action of a Bier block. One study reported that local anesthetics act on major nerve trunks, while two other studies indicated that the main site of action of the local anesthetic in a Bier block is on the smaller nerves and possibly the sensory nerve endings.^21^, ^22^ Due to the complex manifestation of CRPS and its nondermatomal involvement, we thought that perhaps the Bier block method, which is effective on small nerve and sensory nerve endings, could reduce the pain of CRPS patients. Our patient underwent a Bier block with lidocaine 0.5% and 8 mg dexamethasone, and she experienced relief from the constant, debilitating pain of CRPS for the six‐month follow‐up period. Moreover, the procedure had a positive impact on the patient's sleep and daily functioning. We think that repeating this block several times at intervals of several months may keep the patient painless for a longer period of time. Due to the complex pathophysiology of CRPS, many studies on the management of these patients are ongoing.^23^, ^24^, ^25^, ^26^ However, there are still many questions about the mechanism and treatment of this syndrome. It is hoped that with the use of various methods of pain reduction, more help can be given to this group of patients. In conclusion, the Bier block was successful in the pain management of CRPS type 1 and had a positive impact on daily functioning, sleep, and the performance of routine activities for a period of several months.

## CONFLICT OF INTEREST

None of the authors have any proprietary interests or conflicts of interest related to this submission.

## AUTHOR CONTRIBUTIONS

Seyed Ali Emami: contributed to concept and design; Hossein Majedi: involved in data acquisition and data analysis/interpretation; Ebrahim Espahbodi: drafted the manuscript; Mehdi Sanatkar: revised the manuscript and involved in supervision; all authors: read and approved the final manuscript.

## ETHICAL STATEMENT

Written informed consents were obtained from our patient.

## Data Availability

The data that support the findings of this study are available from the corresponding author upon reasonable request.

## References

[1] Wager J , Brehmer H , Hirschfeld G , Zernikow B . Psychological distress and stressful life events in pediatric complex regional pain syndrome. Pain Res Manag. 2015;20(4):189‐194. 10.1155/2015/139329 26035287 PMC4532204

[2] Birklein F , Dimova V . Complex regional pain syndrome–up‐to‐date. Pain Rep. 2017;2(6):e624. 29392238 10.1097/PR9.0000000000000624 PMC5741324

[3] Harden RN , Oaklander AL , Burton AW , et al. Complex regional pain syndrome: practical diagnostic and treatment guidelines, 4th Edition. Pain Med. 2013;14(2):180‐229 23331950 10.1111/pme.12033

[4] Todorova J , Dantchev N , Petrova G . Complex regional pain syndrome acceptance and the alternative denominations in the medical literature. Med Princ Pract. 2013;22(3):295‐300. 23171669 10.1159/000343905 PMC5586735

[5] Reimer M , Rempe T , Diedrichs C , Baron R , Gierthmühlen J . Sensitization of the nociceptive system in complex regional pain syndrome. PLoS One. 2016;11(5):e0154553. 27149519 10.1371/journal.pone.0154553 PMC4858201

[6] Harden NR , Bruehl S , Perez RSGM , et al. Validation of proposed diagnostic criteria (the “Budapest Criteria”) for Complex Regional Pain Syndrome. Pain. 2010;150(2):268‐274. 20493633 10.1016/j.pain.2010.04.030 PMC2914601

[7] Kim ED , Yoo WJ , Kim YN , Park HJ . Ultrasound‐guided pulsed radiofrequency treatment of the cervical sympathetic chain for complex regional pain syndrome. Medicine. 2017;96(1):e5856. 28072749 10.1097/MD.0000000000005856 PMC5228709

[8] Goh EL , Chidambaram S , Ma D . Complex regional pain syndrome: a recent update. Burns Trauma. 2017;5:2. 28127572 10.1186/s41038-016-0066-4 PMC5244710

[9] Shim H , Rose J , Halle S , Shekane P . Complex regional pain syndrome: a narrative review for the practising clinician. Br J Anaesth. 2019;123(2):e424‐e433. 31056241 10.1016/j.bja.2019.03.030 PMC6676230

[10] Shaofeng PU , Chen J , Xing GU , et al. Effects of ultrasound‐guided stellate ganglion block on cervical vascular blood flow: study protocol for a randomized controlled trial. Trials. 2018;19:426. 30086776 10.1186/s13063-018-2736-y PMC6081863

[11] Ding Y , Yao P , Li H , Zhao R , Zhao G . Evaluation of combined radiofrequency and chemical blockade of multi‐segmental lumbar sympathetic ganglia in painful diabetic peripheral neuropathy. J Pain Res. 2018;11:1375‐1382. 30100752 10.2147/JPR.S175514 PMC6067610

[12] Patel RH , Sheth R , Hus N . Complex regional pain syndrome caused by an axillary lipoma. Cureus. 2020;12(12):e12280. 33510987 10.7759/cureus.12280 PMC7828746

[13] Gierthmühlen J , Baron R , Blankenburg M , Zernikow B , Maier C . Spontaneous recurrent episodes of wrist pain in a 16‐year‐old girl: a case of complex regional pain syndrome. Pain Rep. 2016;1(5):e578. 29392198 10.1097/PR9.0000000000000578 PMC5770168

[14] Maiarù M , Morgan OB , Tochiki KK , et al. Complex regulation of the regulator of synaptic plasticity histone deacetylase 2 in the rodent dorsal horn after peripheral injury. J Neurochem. 2016;138(2):222‐232. 26998823 10.1111/jnc.13621 PMC4982040

[15] Eldufani J , Elahmer N , Blaise G . A medical mystery of complex regional pain syndrome. Heliyon. 2020;6(2):e03329. 32149194 10.1016/j.heliyon.2020.e03329 PMC7033333

[16] Bharwani KD , Dik WA , Dirckx M , Huygen FJPM . Highlighting the role of biomarkers of inflammation in the diagnosis and management of complex regional pain syndrome. Mol Diagn Ther. 2019;23(5):615‐626. 31363934 10.1007/s40291-019-00417-x PMC6775035

[17] Ferrillo MG . Treatment of complex regional pain syndrome with stellate ganglion local anesthetic blockade: a case report of one patient’s experiences with traditional bupivacaine HCl and liposome bupivacaine. Clinical Case Rep. 2016;4(9):861‐865. 10.1002/ccr3.614 PMC5018589 27648263

[18] Patel P , Thadeshwar S , Maru M , Desai R , Fahey J . Reflex sympathetic dystrophy of the right hand following an acute traumatic injury. Cureus. 2019;11(8):e5363. 31608198 10.7759/cureus.5363 PMC6783201

[19] Wong GY , Wilson PR . Classification of complex regional pain syndromes. New concepts. Hand Clin. 1997;13:319‐325. 9279537

[20] Kingery WS . A critical review of controlled clinical trials for peripheral neuropathic pain and complex regional pain syndromes. Pain. 1997;73:123‐139. 9415498 10.1016/S0304-3959(97)00049-3

[21] Fallatah SMA . Successful management of complex regional pain syndrome type 1 using single injection interscalene brachial plexus block. Saudi J Anaesth. 2014;8(4):559‐561. 25422619 10.4103/1658-354X.140903 PMC4236948

[22] Misidou C , Papagoras C . Complex regional pain syndrome: an update. Mediterr J Rheumatol. 2019;30(1):16‐25. 32185338 10.31138/mjr.30.1.16 PMC7045919

[23] Bar‐Shalita T , Livshitz A , Levin‐Meltz Y , Rand D , Deutsch L , Vatine J‐J . Sensory modulation dysfunction is associated with complex regional pain syndrome. PLoS One. 2018;13(8):e0201354. 30091986 10.1371/journal.pone.0201354 PMC6084887

[24] Birklein F , Ajit SK , Goebel A , Perez RSGM , Sommer C . Complex regional pain syndrome — phenotypic characteristics and potential biomarkers. Nat Rev Neurol. 2018;14(5):272‐284. 29545626 10.1038/nrneurol.2018.20 PMC6534418

[25] Halicka M , Vittersø AD , Proulx MJ , Bultitude JH . Neuropsychological changes in complex regional pain syndrome (CRPS). Behav Neurol. 2020;2020:4561831. 32399082 10.1155/2020/4561831 PMC7201816

[26] Chang C , Patrick McDonnell M , Gershwin E . Complex regional pain syndrome – Autoimmune or functional neurologic syndrome. J Transl Autoimmun. 2021;4:100080. 33490941 10.1016/j.jtauto.2020.100080 PMC7804982

